# Strategies for Biomaterial-Based Spinal Cord Injury Repair *via* the TLR4-NF-κB Signaling Pathway

**DOI:** 10.3389/fbioe.2021.813169

**Published:** 2022-04-29

**Authors:** Bin Lv, Naiting Shen, Zhangrong Cheng, Yuhang Chen, Hua Ding, Jishan Yuan, Kangchen Zhao, Yukun Zhang

**Affiliations:** ^1^ Department of Orthopaedics, Union Hospital, Tongji Medical College, Huazhong University of Science and Technology, Wuhan, China; ^2^ Tongji Hospital, Tongji Medical College, Huazhong University of Science and Technology, Wuhan, China; ^3^ Department of Orthopedics, Affiliated People’s Hospital of Jiangsu University, Zhenjiang, China

**Keywords:** biomaterial, spinal cord injury, autophagy, TLR4-NF-κB, signaling pathway

## Abstract

The repair and motor functional recovery after spinal cord injury (SCI) has remained a clinical challenge. Injury-induced gliosis and inflammation lead to a physical barrier and an extremely inhibitory microenvironment, which in turn hinders the recovery of SCI. TLR4-NF-κB is a classic implant-related innate immunomodulation signaling pathway and part of numerous biomaterial-based treatment strategies for SCI. Numerous experimental studies have demonstrated that the regulation of TLR4-NF-κB signaling pathway plays an important role in the alleviation of inflammatory responses, the modulation of autophagy, apoptosis and ferroptosis, and the enhancement of anti-oxidative effect post-SCI. An increasing number of novel biomaterials have been fabricated as scaffolds and carriers, loaded with phytochemicals and drugs, to inhibit the progression of SCI through regulation of TLR4-NF-κB. This review summarizes the empirical strategies for the recovery after SCI through individual or composite biomaterials that mediate the TLR4-NF-κB signaling pathway.

## 1 Introduction

Spinal cord injury (SCI) is one of the most fatal diseases, resulting in disabilities and sequelae for individuals (Wang et al., 2021a). The pathophysiology of SCI is complex but well established. Long-term complications of secondary injury after SCI, including inflammation, autophagy, apoptosis, and glial scar formation, create an inhibitory microenvironment that forms a permanent physical and chemical barrier for neuron regeneration and thus requires comprehensive mitigation (Yu and Gu, 2019; Wang et al., 2021a). Here, we highlight the regulation of macrophage/microglia-mediated inflammatory responses activated by the TLR4-NF-κB signaling pathway. This review addresses the mechanisms of pharmacological treatment of SCI through the TLR4-NF-κB signaling pathway, especially based on biomaterials.

As generally known, Toll-like receptors (TLRs) are a family of transmembranous pattern recognition receptors associated with host immune system (Kasurinen et al., 2019). The activation of TLR induces the production of pro-inflammatory cytokines and chemokines, playing a crucial role in innate and adaptive immune function (Chen et al., 2018). Nuclear factor kappa-B (NF-κB) is an important nuclear transcription factor which is located at the hub of the downstream signaling pathway of TLRs. When stimulated by TLRs, NF-κB enters the nucleus and combines with DNA specific sequence to regulate the transcription of genes like inflammatory cytokines and cell anti-apoptosis, thus participates in the specific regulation of inflammation cascade and the precise control of the inflammatory response, mitigating the inhibitory inflammatory microenvironment after SCI repair (Xing et al., 2021). Based on the regulatory function of the TLR4-NF-κB signaling pathway on injury-induced inflammatory response, many drugs target TLR4 to control the inflammation after SCI. Some medicinal products and herbal medicines have been implicated to regulate macrophage/microglia polarization, oxidative stress, and apoptosis *via* the modulation of TLR4 signaling, thus fostering the recovery of injured spinal cord. Some herbal molecules, such as curcumin, ginsenoside Rb1 (GRb1) and catalpol, having various pharmacological effects such as anti-oxidant and anti-inflammatory, are potential immune regulatory factors which can achieve anti-inflammatory effects by inhibiting the activation of TLR4 (Chen et al., 2018). Therefore, these nature molecules are considered as a promising drug resource for the treatment of neural inflammation and the functional restoration of injured spinal cord. Unfortunately, the utilization of these natural products in clinics is limited due to their poor stability and unsatisfying solubility (Lam et al., 2018). Therefore, in order to enhance the stability and solubility of natural small molecules, scientists pay much attention to biomaterial-based drug delivery systems.

Numerous new biomaterials loaded with drug complexes that target TLR4 have successfully been designed in the laboratory for inflammatory alleviation and regulation of autophagy and apoptosis *via* the TLR4-NF-κB signaling pathway. However, biomaterials as drug delivers may result in low loading efficacy and poor biodegradability and biocompatibility (Xing et al., 2021). Furthermore, the TLR4-NF-κB signaling pathway is a crucial pathway through which specific drugs act to mitigate the inflammatory microenvironment (Saleh et al., 2021). Biomaterials that exhibit over-expression or under-expression of the TLR4-NF-κB signaling pathway can poorly control local inflammatory response, leading to an inability to effectively inhibit the inflammatory microenvironment (Lin et al., 2014). Therefore, the TLR4-NF-κB pathway ought to be considered as an important link in the mechanism of biomaterial drug delivery. Satisfying biocompatibility and regulation of the TLR4-NF-κB signaling pathway could exert important effects on the research and design of biomaterials. Many studies and experiments, such as rhein hydrogels (Zheng et al., 2019), have suggested that hydrogel, natural materials and nano-morphology can be modified and assembled with targeted molecules to achieve satisfying anti-inflammatory effects by regulating the TLR4-NF-κB pathway (Zheng et al., 2019; Li et al., 2021a). A substantial body of innovative work has been done on the direct self-assembly of biomaterials with small molecules. Many assembly systems (e.g. rhein, paclitaxel, and dexamethasone) have been developed after the structural modification of natural products (Wang et al., 2012; Tang et al., 2017; Zheng et al., 2019). Moreover, in addition to drug delivery, natural biomaterials are good vectors for neural stem cells (NSCs) transplantation and nerve regeneration because they are less likely to induce immune responses. Composite biomaterials may have synergistic effects.

Previous reviews have demonstrated the crucial role of TLR4-NF-κB signaling pathway after SCI, while the relevant appropriate biomaterials that assembled with herbal molecules or stem cells (SCs) and act on TLR4-NF-κB pathway have not been systematically summarized. The present review focuses on the specific mechanisms that affect SCI repair through the TLR4-NF-κB signaling pathway, including macrophage/microglia mediated inflammation, autophagy, apoptosis and oxidative stress. And we also describe the types and design of biomaterials for applications of neuron regeneration and tissue repair, and suggest future applications of biomaterials that act on signaling mechanisms.

## 2 TLR4 Signaling

TLRs are a family of transmembrane receptors with a cytoplasmic signaling domain called Toll/Interleukin 1 receptor (IL-1R) domain (Chen et al., 2018; Zhi et al., 2021; Jiang and Zhang, 2021; Saleh et al., 2021). TLRs can identify pathogen-associated molecular patterns (PAMPs) or damage-related molecular patterns (DAMPs) to detect pathogens (Chen et al., 2018). When activated by pathogens, cytokines or stress induced in cells (Rahimifard et al., 2017; Abbaszadeh et al., 2020), TLRs in turn activate different types of genes that regulate host defense, such as chemokines, inflammatory cytokines, microglia and myosin heavy chains to initiate different immune response cascades (Sato et al., 2020). TLRs in mammals can also induce a variety of effector molecules, including inducible nitric oxide synthase (iNOS) and antimicrobial peptides which kill microbial pathogens (Van Gorp et al., 2019). TLRs activate NF-κB and mitogen-activated protein kinases (MAPK) based on TIR domains, inducing target genes (Takeda and Akira, 2005; Akira et al., 2006). NF-κB is a transcriptional activator downstream of TLR4 and plays a crucial role in up-regulation of pro-inflammatory cytokine. Prior research on neurodegenerative diseases have demonstrated that NF-κB is a key signal transducer in inflammatory regulation of neurons and microglia (Stone et al., 2011). When activated in microglia, NF-κB up-regulates reactive oxygen species (ROS) and secretion of tumor necrosis factor-β (TNF-β), interleukin-1 (IL-1) and interferon-β (IFN-β), resulting in secondary neurotoxic effects (Block et al., 2007). Meanwhile, NF-κB can exert important effects on maintaining tumor necrosis factor-α (TNF-α) homeostasis, and the NF-κB-TNF-α interactions are bidirectional. The activation of NF-κB up-regulates the expression of TNF-α. Whereas, when NF-κB binds to the DICER promoter, it inhibits TNF-α expression by producing mature forms of miR-125b and miR-130a (Guan et al., 2015). TNF-α plays an important role in immunity, enhancing CD40, CD80, CD83, and CD86 expression and acting on TNF-α receptor 1 (TNFR1)-associated death domain protein (TRADD)-TNFR-associated factor 2 (TRAF2)-NF-κB signaling pathway. For example, up-regulation of TNF-α in bone marrow dendritic cells (DCs) enhanced DCs induced T cell differentiation (Li et al., 2015). Paeoniflorin-6′-O-benzene sulfonate (CP-25) inhibits the TRADD-TRAF2-NF-κB signaling pathway *via* TNF-α, thereby inhibiting DCs functions (Li et al., 2015).

Among the TLR family, TLR4 is the first TLR known to recognize and respond to pathogen-associated molecular patterns including lipopolysaccharides (LPS) on plasma membranes (El-Obeid et al., 2020; Splichal et al., 2020). It has been reported that intraspinal injection of LPS can up-regulate the expression of TLR4, producing inflammatory cytokines (Li et al., 2017; Reuschel et al., 2019). In humans, the main cells expressing TLR4 are of myeloid origin. In the central nervous system (CNS), TLR4 is most abundant in microglia (Karimy et al., 2020). Besides, TLR4 is also expressed in other myeloid cells, neurons, astrocytes, B cells, endothelial cells and epithelial cells, regulated by several cytokines (He et al., 2019; He et al., 2020). When binding to LPS, TLR4 dimerizes and induces one or more of four intracellular adaptor proteins: TIR domain-containing adapter molecule 1/2, MyD88, TIR domain-containing adaptor protein (TIRAP) (Chen et al., 2018), and in turn triggers two standard signaling cascade models: myeloid differentiation primary response gene 88 (MyD88)-dependent pathway and IL-1R domain-containing adaptor inducing-β interferon (TRIF)-dependent pathway (Latif et al., 2020; Saleh et al., 2021).

MyD88-dependent pathway derives from the cytoplasmic TIR domain (Guven-Maiorov et al., 2015). The activation of MyD88 elicits automatic phosphorylation of IL-1 receptor-associated kinase (IRAK) (Saleh et al., 2021). They temporally bind to IRAKs/TRAF6 complex and transformation factor-β-activated kinase 1 (TAK1)/TAK1-binding proteins (TABs) complex, activating IκB kinase (IKK) and MAPK, namely extracellular signal-regulated kinase 1/2 (ERK1/2), c-Jun N-terminal kinase (JNK), p38 (Takeda and Akira, 2005; Parveen et al., 2021). NF-κB, as a transcription and regulatory factor binding to IκB, which can be phosphorylated by ROS, macrophages and inflammatory cytokines (Meylan et al., 2004; Xing et al., 2021), is in turn activated to induce the transcription of the activator protein-1 (AP-1), and subsequently stimulates production of pro-inflammatory factors and free radicals (Newton and Dixit, 2012; Karimy et al., 2020; Zhi et al., 2021). In the MyD88-independent pathway, TLR4 mediates the late activation of NF-κB through recruitment of TRIF and translocating chain-associated membrane protein (TRAM) containing TIR domains (Saleh et al., 2021). TRIF leads to the ubiquitination of TRAF3, which in turn induces the binding interaction between the downstream protein kinase TBK1 and IκB kinase epsilon (IKKϵ) (Sato et al., 2003; Meylan et al., 2004; Takeda and Akira, 2005). Thereafter, the TBK1-IKKϵ complex phosphorylates and activates the interferon regulatory factor 3 (IRF3), a transcription factor inducing IFN-I production, and ultimately drives the expression of IFN-β, inducing interferon inducible protein 10 (IP-10), monocyte chemoattractant protein 5 (MCP-5) and iNOS (Sato et al., 2003; Saleh et al., 2021).

### 2.1 SCI and the Mechanism of TLR4-NF-κB Signaling Pathway

**FIGURE 1:**
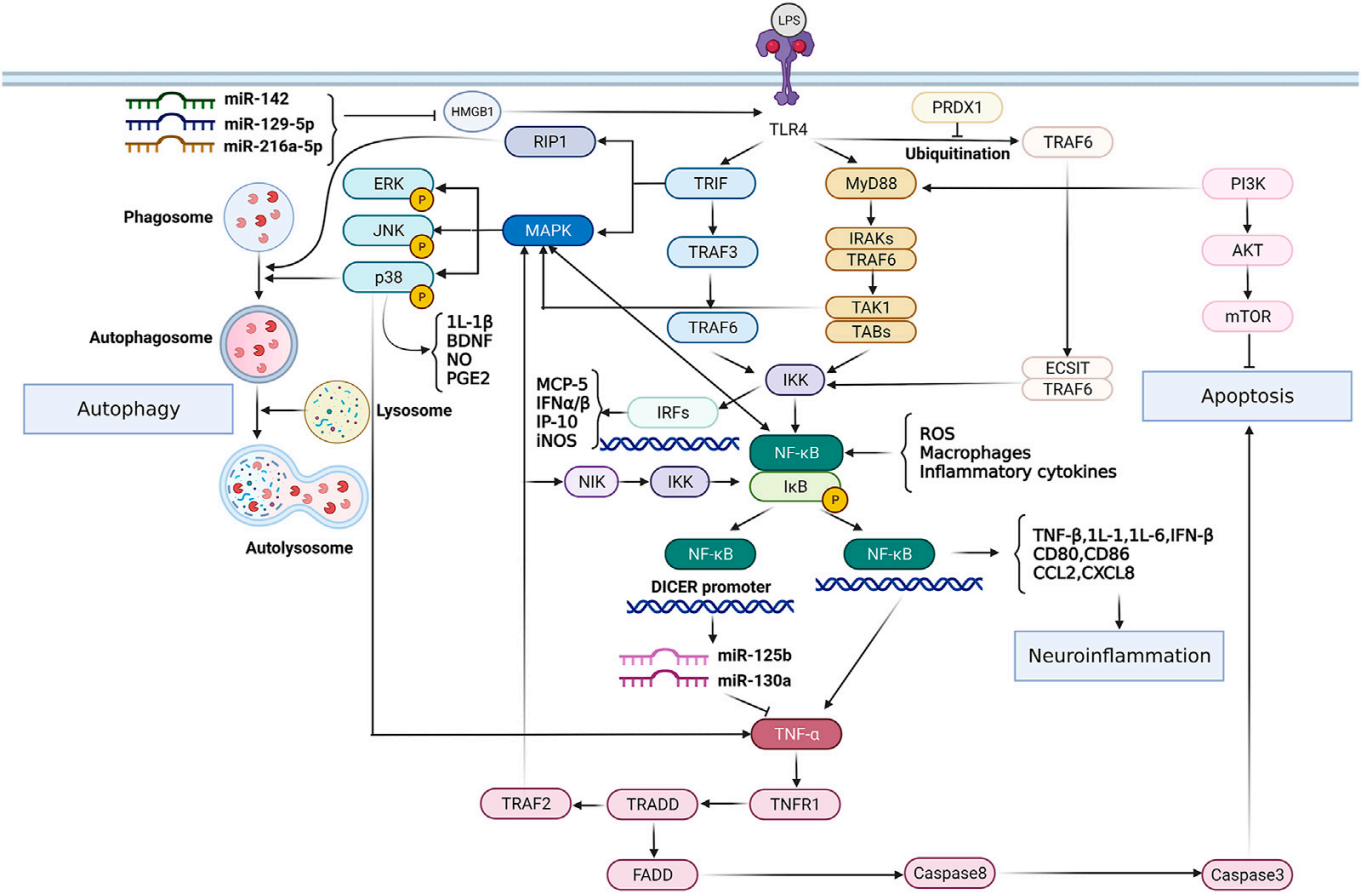
The role of TLR4-NF-κB signaling pathway in modulating inflammation, autophagy and apoptosis. TLR4 activates transcription factor NF-κB through MyD88-dependent/TRIF-dependent signaling pathways respectively, thus up-regulating the expression of inflammatory mediators and chemokines, and exacerbating neuroinflammation. However, the TLR4-NF-κB signaling pathway can be inhibited by certain kinds of miRNAs. In addition, NF-κB imparts reciprocal regulation with TNF-α. Activated NF-κB regulates TNF-α homeostasis, and TNF-α in turn activates NF-κB via TRADD/TRAF2/MAPK signaling pathway and promotes apoptosis through TRADD/FADD/caspase8 pathway. On the other hand, the TLR4/MAPK-P38 pathway plays an important role in the regulation of inflammation and autophagy, eliciting innate immune responses.

According to the pathologic process, SCI can be grouped into primary injury and secondary injury (Xing et al., 2021). The primary injury is instantaneous and irreversible. The secondary injury occurs on the basis of primary injury and reversibly runs through the whole course of SCI (Kiyotake et al., 2020). It includes demyelination, axon and neuron necrosis and cellular tissue damage (Xing et al., 2021; Kiyotake et al., 2020; Berry et al., 2016). Neurons are highly specialized for intercellular communication through axons and dendrites in the spinal cord of CNS, thus it’s crucial to maintain neuronal integrity throughout their life cycle (Lisek et al., 2020). Traumatic impairment of normal function of central and peripheral axons results in molecular and progressive morphological changes in stress response (Schellino et al., 2019). The neuron triggers granular degeneration and cytoskeletal disintegration of axon distal to lesion sites (Schellino et al., 2019). Axonal injury can induce neuronal apoptosis and axonal degeneration under different traumatic conditions. In addition, CNS injury is often accompanied by microglia/macrophage-mediated inflammatory responses (Zhang et al., 2020a). Neuroinflammation during neurodegeneration and demyelination results in oxidative stress, chronic microglial activation, mitochondrial damage and axon damage (Mihai et al., 2019). The TLR4-NF-κB signaling pathway plays an important role in these processes. It has been demonstrated that TLRs are effective activators of inflammatory responses in different pathological conditions of spinal cord. When activated, TLRs can induce the production of IFNs, cytokines, chemokines and NF-κB (Newton and Dixit, 2012; Sato et al., 2003). For instance, activation of the TLR4-mediated NF-κB/IL-1β aggravates inflammatory damage in the blood-spinal barrier (BSCB) and nerve cells after spinal ischemia reperfusion (Li et al., 2017). Figure 1 indicates the role of TLR4-NF-κB signaling pathway in regulating inflammation, autophagy and apoptosis. Activated TLR4 in spinal cord, in addition to these common features, also promotes iron uptake and storage, causing iron mediated progressive injury to neurons and oligodendrocytes and thereby aggravating their loss (Goldstein et al., 2017). However, TLR4 does not consistently play a negative role in all kinds of neurons. It has been evinced that LPS-activated TLR4-NF-κB signaling pathway may increase the survival of intestinal neurons in mice, and therefore, TLR4 is involved in regulating intestinal homeostasis and promoting gastrointestinal motility (Anitha et al., 2012). In addition, activation of TLR4 has been reported to promote self-renewal of human neural stem cells (hNSCs) *in vivo*. In amyotrophic lateral sclerosis (ALS) rats or immunodeficient mice transplanted with hNSCs, endogenous expression of TLR4 is consistent maintained, thereby independent of neuroinflammation (Grasselli et al., 2018). Grasselli et al. demonstrested that LPS activated TLR4 signaling is essential for the proliferation and differentiation of hNSCs, which is of significance for the study of neurogenesis (Grasselli et al., 2018). Nevertheless, the inhibitory effect of TLR4-NF-κB signaling pathway on neuronal regeneration and differentiation in the pathological mechanisms of SCI is still dominant.

#### 2.1.1 Microglia and Inflammation

Microglia/macrophage-mediated inflammatory responses often result in self-destructive lesions or necrosis of the surrounding tissue, which are the key factors leading to secondary injury (Bi et al., 2017a; Xia et al., 2020). After SCI, macrophages are induced to polarize by various cytokines and bioactive substances that are often produced in inflammatory responses. Macrophages with the M0 phenotype exist widely in various organs and tissues including brain (Microglia) and liver (Kupffer cells) (Xing et al., 2021). When stimulated by IFN-γ, LPS, TNF-α, macrophages polarize into M1-phenotype macrophages, which act as pro-inflammation. Pro-inflammatory cytokines produced by M1 macrophages (including IL-1β, IL-6 and TNF-α) up-regulate nicotinamide adenine dinucleotide phosphate (NADPH) and ROS, thereby inducing pathogen clearance and tissue damage (Shapouri-Moghaddam et al., 2018; Ren et al., 2019). IL-1β and IL-6 produced by M1 are associated with LPS-induced inflammation and local tissue damage such as chronic bronchitis and acute lung injury (Asgharpour et al., 2019). When stimulated by IL-4, IL-10, IL-13, colony-stimulating factor 1 and transforming growth factor-β (TGF-β), M2 macrophages exert anti-inflammatory effects in the treatment of SCI by producing anti-inflammatory cytokine IL-10. IL-10 promotes tissue repair through secretion of arginase-1 against iNOS activity, thus inducing M2 macrophage polarization (Asgharpour et al., 2019).

Within CNS, microglia and astrocytes are macrophages involved in the cellular immune process of the nervous system and regulation of neuroinflammation (Rojewska et al., 2018). Classically, M1 microglia cause severe inflammatory responses and inhibit myelin regeneration through release of inflammatory factors, cytotoxic agents and free radicals, such as colony stimulating factor, TNF-α, IL-1 and IL-6. In contrast, M2 microglia exerts anti-inflammatory effects. M2a and M2c release anti-inflammatory factors such as IL-4 and IL-13 to engulf the debris of damaged nerve cells for the repair and regeneration of injured nerve cells and tissues. Additionally, M2 microglia can inhibit chondroitin sulfate proteoglycos (CSPGs)-induced axonal degeneration and clear myelin debris for remyelination to proceed. 3–10 days after demyelination, M2 microglia and macrophages regulate the phagocytosis of degenerated myelin through the cAMP cascade, promoting the generation of new myelin sheath and creating a more favorable microenvironment for neurons (Mestre et al., 2015). Therefore, regulation of the polarization of M1/M2 macrophages is a new direction for inhibition of M1 macrophage-induced pro-inflammatory injury and promotion of neural repair in post-SCI treatment.

Among numerous receptors expressed on microglia, TLRs (especially TLR2, TLR3, and TLR4) illustrates a connection between microglial activation and nerve injury, showcasing a crucial role of microglia in neuropathy (Schütze et al., 2012; Lim et al., 2013). In response to different CNS stimuli post-SCI, TLR2, TLR3 and TLR4, as molecular receptors linked by pathogens, induce activation of spinal cord glia (astrocytes and microglia) and enhance inflammatory cascades (Buchanan et al., 2010). Rats knocked out TLR2 or TLR4 have been demonstrated to have less activated microglia and fewer symptoms of neuropathic pain after nerve injury such as SCI (Piao et al., 2018; Yang et al., 2020). It is well known that TLR4 up-regulates inflammatory cytokines *via* MyD88/TRIF-dependent signaling pathways. Myd88-dependent TLR signaling is associated with the phosphorylation of SNAP-23 on dendritic cell phagosomes (Nair-Gupta et al., 2014), which stabilizes SNARE complexes. SNARE complexes fuse with endosomes and ultimately elicit cross-presentation. The TLR/MyD88/NF-κB pathway in microglia may contribute to the reduction of SNAP-23. In contrast to astrocytes, microglia has higher TLR expression and stronger response to LPS, a well-known microglia activator by activating TLR4 (Harry, 2013). In addition, the functional TLR4 in microglia is essential for the response of astrocytes to TLR2 agonists (Facci et al., 2018). LPS induces the synthesis of relevant TLR2 by activating TLR4. Activated microglia show elevated expression of the microglial markers CD11b and ionized calcium-binding adaptor molecule 1, and increased phosphorylation of p38 MAPK, which can be reduced by the antisense knockout of spinal TLR3 and TLR4 (Liu et al., 2012). Subsequently, the activation of P38 in spinal microglia leads to up-regulation of pro-inflammatory cytokines including IL-1β, TNF-α, brain-derived neurotrophic factor (BDNF), NO and prostaglandin E2 (PGE2) (Ji and Suter, 2007; Ji et al., 2009). These microglial mediators can effectively modulate synaptic transmission by enhancing the excitability of spinal cord neurons and inhibiting inhibitory synaptic transmission (Liu et al., 2012). TLR4 regulates the release of IL-1β, TNF-α, BDNF, NO and PGE2 through activating p38 MAPK in microglia. Therefore, drugs or herbal molecules such as BoNT/A and GinsenosideRb1 (GRb1) play an important anti-inflammatory role *via* inhibition of TLR4-NF-κB signaling pathway in microglia. In addition, microglia-induced neuroinflammation also activates the TLR4-NF-κB signaling pathway through high-mobility group box 1 (HMGB1) or TNFR1-TRADD-TRAF2-NF-κB pathway through TNF-α (Shi et al., 2018a). While arctigenin, a lignan-derived bioactive element of Fructus Arctii, can inhibit the binding of HMGB1 toTLR4 and TNF-α to TNFR1 simultaneously, thereby suppressing microglia activation and neuroinflammation (Gao et al., 2020a; Xu et al., 2020). In the CNS, TLR4 as an inflammatory mediator is not only expressed in microglia, but also plays an important role in other types of myeloid cells, especially through myD88-dependent signaling pathways. TLR4 oligomerizes and binds to TIRAP through TIR-TIR domain interaction. This promotes the recruitment of additional MyD88 molecules, leading to downstream activation of TAK1. IKKα, IKKβ, and IKKγ complexes activate NF-κB through phosphorylation of IκB, ultimately up-regulating the expression of pro-inflammatory cytokines.

#### 2.1.2 Autophagy

Autophagy is regarded as a crucial anti-inflammatory mechanism that can prevent intravascular damage resulting from various endogenous or infectious sources and keep off excessive or unnecessary inflammation (Deretic and Levine, 2018). A significant increase in autophagy markers LC3-II and Beclin-1 manifests the up-regulation of autophagy levels (Lee and Lee, 2016). The alteration of autophagy flux after SCI depends on the severity and site of injury (Lipinski et al., 2015). The enhancement of autophagy flux after moderate injury may be protective, playing a positive role in coping with stress post-SCI. Autophagy can stabilize microtubules through degradating superior cervical ganglion protein 10 to facilitate axon growth, and protect neurons from endoplasmic reticulum stress, all of which are conducive to neuronal survival and functional recovery (Shi et al., 2021). Liu et al. have revealed that induction of autophagy flux in astrocytes is beneficial in promoting endogenous neuroprotection and neurological recovery (Liu et al., 2018a). However, suppression of autophagy flux after severe injury can elicit neuronal cell death (Zhou et al., 2017). As an important degradation pathway in the secondary stage of SCI (Zhou et al., 2021), autophagy modulates the nervous system homeostasis and immune inflammation by regulating innate immune signaling pathways including microglia polarization (Jin et al., 2018; Li et al., 2021b). Su et al. revealed that autophagy inhibition is involved in excessive pro-inflammatory responses in microglia or brain macrophages (Ye et al., 2020). Although the underlying signaling mechanisms of autophagy regulating microglial inflammation and the relief of autophagy-mediated inhibition of inflammation both remain unclear, numerous studies have shown that the activation of autophagy on microglia after SCI changes with time and the number of microglia. Excessive activation of macrophage also results in inflammatory responses and aggravates SCI. He et al. found that LPS-induced p38MAPK-dependent phosphorylation of uncoordinated 51-like kinase 1 (ULK1) in microglia reduces the autophagy flux and inhibits the immunosuppressive activity of autophagy in response to stimulation, enabling activated microglia to fully induce inflammation (Wang et al., 2017a).

The signaling pathways controlling autophagy are complex. Mammalian target of rapamycin (mTOR), a multifunctional serine/threonine protein kinase, is an important therapeutic target for regulation of microglial autophagy. The phosphatidylinositol 3-kinase (PI3K)/Protein Kinase B (AKT)/mTOR signaling pathway, a major pathway regulating autophagy (Fu et al., 2010), is a major upstream regulator of autophagy and mechanistic target of rapamycin complex 1 (mTORC1) is a key negative regulator (Fu et al., 2010; Cuadrado et al., 2019). Based on a series of *in vitro* and *in vivo* studies, Zhang et al. revealed that the inhibition of PI3K/AKT/mTOR pathway can enhance microglial autophagy, thereby increasing M2-type polarization of microglia (Zhang et al., 2021). The PM-derived exosomes (PM-Exos) based on PI3K/AKT/mTOR signaling promotes autophagy by inhibition of AKT and mTOR expression, triggering the anti-inflammatory function of local microglia, which is an important avenue in neuronal protection (Zhang et al., 2021). Furthermore, AMP-activated protein kinase (AMPK)/mTOR signaling pathway also participates in regulation of autophagy-inflammation pathway. Together with AMPK, mTOR initiates autophagy *via* activation of ULK1. Autophagy can in turn suppress the activation of inflammasome to inhibit inflammatory responses. Pien-Tze-Huang (PTH), a Chinese medicine, promotes autophagy and inhibits microglial activation through the AMPK/mTOR/ULK1 pathway, thereby suppressing neuroinflammation mediated by nucleotide-binding oligomerization domain, leucine-rich repeat-, and pyrin domain-containing 3 (NLRP3) inflammasome (Huang et al., 2021). Salidroside, a plant-based extract, also modulates microglial autophagy flux *via* AMPK/mTOR pathway (Gu et al., 2020). Therefore, certain Chinese medicines including PTH, salidroside and resveratrol, can play a neuroprotective role through the autophagy-inflammation pathway (Xu et al., 2018), implicating the autophagy-inflammation pathway as a new direction for therapeutics of SCI.

Accumulating evidence has demonstrated the crosstalk between autophagy and TLRs signaling. Different TLRs may trigger autophagy through synergistic or independent activation mechanisms. For instance, the overexpression of TLR4 is inhibited by AMPK phosphorylation, which thereby suppresses the TLR4/MAPK/NF-κB signaling pathway to induce autophagy, whereas TLR2 takes effect through Lyn and NF-κB (Tao et al., 2017). Other signaling pathways including plasminogen activator inhibitor 2 (PAI2), PI3K/AKT and MyD88/NF-κB are associated with both TLR2 and TLR4 (Shi and Kehrl, 2008). Shu et al. found that *Streptococcus pneumoniae* protein PepO enhances the phagocytic function of macrophages by interacting with TLR2 and TLR4 respectively, but the underlying mechanism remains unclear (Shu et al., 2020). Further studies showed that autophagy is involved in the enhancement of phagocytic function of macrophages. Pepo manipulates the TLR2/4-dependent PI3K/AKT signaling pathway, which is a main regulatory axis of autophagy regulation (Zhang et al., 2016), to regulate macrophage autophagy and thereby enhance macrophage phagocytosis. Activation of TLR2/4 can trigger a signaling cascade in which MAPK-ERK, p38 and JNK are activated, leading to secretion of pro-inflammatory factors mediated by phosphorylation of MAPK, NF-κB and IRFs (Medzhitov, 2007). Meanwhile, during TLRs signal transduction, PI3K can bind to receptor or adapter molecules TIRAP or MyD88, resulting in the activation of its downstream targets NF-κB and AKT (Akira and Takeda, 2004; Santos-Sierra et al., 2009).

Autophagy can also modulate the phenotypic changes of microglia *via* NF-κB pathway (Fu et al., 2010). TLR4-NF-κB signaling pathway exerts important effects on the regulation of the autophagy-inflammation pathway. Autophagy-related 16-like 1-deficient macrophages secrete large amounts of inflammatory cytokines IL-1β and IL-18 after stimulation of TLR4 ligands such as LPS (Hampe et al., 2007; Levine and Deretic, 2007). The pretreatment tetracycline decreases TNF-α and IL-6 expression through the NF-κB signaling pathway, inhibiting the activation and phagocytosis of microglia. In addition, the autophagy inhibitor 3-MA also suppresses the expression of its downstream genes by inhibiting the NF-κB pathway (Jiang et al., 2012). The fusion of phagosomes containing TLR ligands with lysosomes in macrophages requires autophagy-related (Sanjuan et al., 2007). The depletion of autophagy proteins LC3B and Beclin-1 leads to dysregulation of mitochondrial accumulation (Mo et al., 2020). Green et al. revealed that damaged mitochondria produce ROS, which results in the activation of NF-κB signaling and inflammasomes, thereby activating caspase-1 up-regulating pro-inflammatory cytokines such as IL-1β, IL-6 and IL-18 (Mo et al., 2020).

Ubiquitin modification, as a key step in autophagy, can be observed in TRAF6, Sequestome 1/p62, BECN1/Beclin1 and other proteins during autophagy (Yan et al., 2020). Studies implicated that autophagy processes ubiquitinate inflammasomes for capture and degradation of inflammasomes, while autophagy blocking increases the expression of IL-1β (Nosaka et al., 2020). Among them, ubiquitination of TRAF6 may be necessary for activating NF-κB and MAPK signaling pathways (Zhu et al., 2019). It has been reported that TRAF6, as a potential target of neuroinflammation, plays an important role in NF-κB activation and autophagy activation induced by TLR4 signal (Min et al., 2018). TRAF6 ubiquitin-ligase activity is highly involved in initiating autophagy. It enables ubiquitination of evolutionarily conserved signaling intermediates in TLRs and Beclin 1, which are respectively required for NF-κB activation and autophagy activation. And activation of autophagy in turn reduces expression of TRAF6 protein in spinal cord. Impaired autophagy may enhance the expression of microglial pro-inflammatory factors and aggravate neuroinflammation through driving the binding of TRAF6 to K63 ubiquitin protein, which up-regulates p38 MAPK and NF-κB. Atorvastatin inhibits the inflammatory activation of BV-2 microglia by inhibiting the TLR4/TRAF6/NF-κB signaling pathway (Han et al., 2018). Similarly, peroxiredoxin 1 (PRDX1) inhibited the activation of NF-κB and autophagy by suppressing the activity of TRAF6 ubiquitin-ligase, thereby inhibiting the bactericidal activity of autophagy (Min et al., 2018). Taken together, TLRs and NF-κB signaling play an important role in the autophagy - inflammation pathway and macrophage phagocytosis. It is a promising treatment protocol for SCI to regulate autophagy and inhibit inflammation with biomaterial-based drugs targeting TLR4-NF-κB signaling pathway.

#### 2.1.3 Apoptosis and Ferroptosis

Apoptosis refers to an orderly and energy-consuming procedure of cell death controlled by genes to maintain a stable internal environment (Beattie et al., 2002). Though the neuronal damage after SCI mainly results from necrosis, apoptosis cannot be negligible. The primary mechanical injury post-SCI activates the apoptotic pathway of the injured cells (Beattie et al., 2002). Within hours to weeks after injury, apoptosis occurs in glial cells and oligodendrocytes of white matter (Beattie et al., 2000; Kuzhandaivel et al., 2011). Sustained damage *via* apoptosis is one of the crucial reasons for the secondary injury post-SCI, and the massive apoptosis of neurons and oligodendrocytes may be responsible for the long-term failure of neurological function recovery after SCI. Therefore, a better understanding of the exact mechanisms of apoptosis initiation following SCI will provide novel strategies for functional recovery.

Apoptosis is initiated by exogenous (death receptors) and endogenous (mitochondria) mediators, which interact with each other in certain signaling parts (Elmore, 2007). The exogenous pathway activates caspase-8 by initiating Fas and TNF-α (Elmore, 2007). Fas/FasL has been evidenced to mediate apoptosis. Upon binding of Fas to CD95/apoptosis antigen-1, Fas associated death domain (FADD) is activated. Subsequently, the binding of TNF-α to TNFR activates TRADD, in turn recruiting FADD (Reed, 2000), and ultimately activating procaspase-8 (Elmore, 2007). The endogenous pathway of apoptosis is characterized by internucleosomal DNA fragmentation, which distinguishes it from necrosis and leads to cell death. The pathway elicits the release of cytochrome C by activating B cell lymphoma 2 (Bcl-2) protein. Pro-apoptotic proteins increase the permeability of mitochondrial membrane, releasing cytochrome C to the cytosol (Abbaszadeh et al., 2020). Cytochrome C forms an apoptosome complex with caspase-9 and apoptotic protease activator -1 (APAF-1), activating other caspases (Garrido et al., 2006). In addition, anti-apoptotic molecules can combine with deoxyadenosine triphosphate (dATP)/APAF-1 to suppress the release of cytochrome C and form a polymer complex activating proaspase-9 (Loreto et al., 2014). Among them, mitochondria play a crucial role in activating apoptosis post-SCI. The loss of mitochondrial function is one of the most remarkable characteristics of secondary injury after SCI. Excessive ROS elicited by SCI widely activate poly (ADP-Ribose) polymerase-1 (PARP-1), thus reducing β-nicotinamide adenine dinucleotide (NAD) and ATP, and releasing mitochondrial apoptosis-inducing factor (AIF) (Sosna et al., 2014), which promotes apoptosis. Experiments showed that PARP-1 protein degradation increased 6 h after SCI and peaked on the third day, and the disappearance is on the 28th day (Gao et al., 2016). However, the molecular mechanisms of programmed cell death following SCI require further elucidation, and certain signaling pathways imply a potentially important role in modulation of apoptosis.

TLR4-NF-κB signaling is a crucial pathway in modulation of apoptosis. In the exogenous pathway of apoptosis, TNF production is caused by LPS, the ligand of TLR4. The binding of LPS and TLR4 leads to secretion of a large amount of immunomotor TNF-α, which activates the innate immune responses through the TLR4-NF-κB or p38 MAPK pathway, playing a kay role in the modulation of apoptosis and inflammation (Hassanein et al., 2021). It has been reported that TNF-α is involved in apoptosis mechanism *via* specific cell surface receptor TNFR 1, inducing apoptosis through TRADD (Gao et al., 2020b). This pathway mediated by TNF α binding to TNFR 1 results from the induction of NF-κB. TRADD, FADD and activatde caspase-8 are responsible for the cleavage of effector caspase-3 and inhibition of receptor-interacting protein kinase 3 (RIPK3)-mixed lineage kinase domain-like (MLKL)-dependent necroptosis (Kondylis et al., 2017). Caspse-3 subsequently triggers apoptosis by translocating the Bcl-family to mitochondria and releasing cytochrome C into the cytoplasm (Abbaszadeh et al., 2020). LPS preconditioning can inhibit NF-κB activation, thereby inhibiting inflammatory responses and promoting neuroprotection, which is associated with the suppression of caspase, thus playing a regulatory role in apoptosis. When NF-κB response is inhibited, the survival friendly ligation of TNFR 1 is transformed into apoptosis-friendly ligation (Dondelinger et al., 2013). Pre-activation of TLR4 is also involved in the inhibition of NF-κB and caspase, mitigating inflammation and up-regulating anti-apoptotic protein signaling pathways. In addition, the crosstalk between IKK/NF-κB and RIPK1 signals plays a crucial role in regulating apoptosis. RIPK1 has been confirmed to have kinase activity-independent functions acting on regulating apoptosis, alleviating inflammation, and maintaining tissue integrity. Takahashi et al. revealed that RIPK1 drives inflammation and cell survival through RIPK3/MLKL-dependent necroptosis and NF-κB, caspase-8-dependent apoptosis (Takahashi et al., 2014). In liver and gut, NF-κB essential modulator inhibits chronic tissue damage and inflammation by regulating apoptosis which is mediated by RIPK1 kinase activity through NF-κB-dependent and independent functions (Kondylis et al., 2017). Cholesterol oxidation products promote apoptosis by lowering Bcl-2 levels, increasing p53 and Bcl-2-associated X protein levels, and activating caspases−8, −9, and −3. A recent study evinced that taxifolin can suppress cell death through the activation of AKT and NF-κB to reduce cholesterol oxidation product-induced neuronal apoptosis (Kim et al., 2017).

Besides, increasing evidence suggested that some miRNAs participate in the pathological process of SCI such as inflammation, oxidation and cell apoptosis *via* the TLR4-NF-κB pathway. Certain Chinese medicines regulate TLR4-NF-κB signaling pathway by affecting miRNAs expression to alleviate the secondary injury to the spinal cord. For example, GRb1, a major component of ginseng, has been confirmed to ameliorate activated microglia-induced neuronal inflammation and inhibit neuronal apoptosis through the miR-130b-5P/TLR4/NF-κB axis (Wang et al., 2021b). Recent studies have also evinced that elevated expression of miR-216a-5p, miR-129-5p and miR-142 can inhibit inflammation and apoptosis *via* HMGB1/TLR4/NF-κB pathway for alleviation of SCI in mice (Wan et al., 2020; Zhenzhen et al., 2021). For instance, catalpol, as an iridoid glycoside extracted from rehmannia glutinosa, suppressed the expression of HMGB1 by up-regulation of miR-142 to hinder TLR4/NF-κB signaling pathway, thereby inhibiting ROS generation, inflammation and apoptosis in microglia BV2 cells (Xia et al., 2020).

Ferroptosis is a new type of nonapoptotic programmed cell death elicited by cell membrane oxidative damage. It is characterized by excessive iron/ROS-dependent lipid peroxidation and dominantly modulated by iron metabolism and coenzyme Q10/glutathione-dependent pathways (Lu et al., 2019; Zhang et al., 2020b). According to models of cultured hippocampal slices of rat organs, ferroptosis was implicated to be the driving factor of neuronal death (Dixon et al., 2012). Recent studies have linked ferroptosis to common diseases involving neuronal injury, such as stroke, Amyotrophic Lateral Sclerosis, Alzheimer’s disease and Parkinson’s disease (Chen et al., 2021; Tan et al., 2021). It has also confirmed that ferroptosis exists and plays an important role in SCI. Zhang et al. found significant changes in ferroptosis markers and in mitochondrial characteristics of ferroptosis in spinal cord tissues of rats post-SCI by transmission electron microscopy (Zhang et al., 2019). SCI was followed by spinal cord hemorrhage, erythrocyte accumulation, rupture, hemolysis, and local iron overload was formed. Besides, oxidative stress activated multitudes of ROS, which intensified the excitatory toxicity of glutamate and thus induced ferroptosis. According to recent studies, ferroptosis might be related to glial scar formation and neuronal death post-SCI. Hao et al. found that the treatment of SCI with deferroamine down-regulated the expression of total iron ion, TNF-α, and caspase-3, suppressing glial scar formation and apoptosis (Hao et al., 2017). These studies implicate that ferroptosis is a new therapeutic target for SCI treatment. Nevertheless, there is still lacking sufficient research illustrating specific mechanism of ferroptosis in SCI.

TLR4 is linked to the regulation of ferroptosis in inflammatory responses. Hypoxia-ischemic injury of hippocampus in neonatal rats can significantly up-regulate TLR4 and p53 levels. Subsequently, activation of TLR4 can induce hippocampal neuronal ferroptosis after hypoxic-ischemic brain damage (HIBD) and oxygen-glucose deprivation (OGD), enhancing the expression of ferropotosis-involved genes. Inhibition of TLR4-P38MAPK signaling pathway can alleviate oxidative stress and mitochondrial damage, inhibit ferroptosis and alleviate neuroinflammation after HIBD, eventually improve the vitality of nerve cells. Intensified autophagy and ferroptosis has been demonstrated to contribute to heart failure, but its underlying mechanisms are still not fully understood. Chen et al. revealed that TLR4/NADPH oxidase 4 (NOX4) pathways can induce the activation of autophagy and ferroptosis in rats after heart failure, suggesting that TLR4 is a promising therapeutic agent for heart failure by retarding autophagy/ferroptosis-mediated cell death (Chen et al., 2019).

#### 2.1.4 Anti-oxidative Effects

Intracellular homeostasis is dominantly maintained by reduction/oxidation homeostasis. Metabolic processes or toxic insults can cause an imbalance between pro-oxidant and anti-oxidant, reaching a state of “oxidative stress”. Oxidative stress, a crucial modulator of common neurodegenerative diseases, may lead to functional deterioration in nerve cell structure such as autophagy, apoptosis and necrosis (Dodson et al., 2013; Tejchman et al., 2021). Secondary injury after traumatic SCI includes oxidative stress and inflammatory cascade, which can aggravate neuronal loss and hinder neuronal regeneration. SCI-induced oxidative stress also inhibits endogenous repair of microvasculature and endothelial functions, thereby impeding the dynamic balance and functional recovery of neural networks through limited nutrient delivery (Peng et al., 2021). This identifies oxidative stress as an important therapeutic target for SCI treatment. Microglia-derived exosomes exert antioxidant effects on spinal microvascular endothelial cells by activating keap1/nuclear factor erythroid 2-related factor 2 (Nrf2)/heme oxygenase 1 (HO-1) signaling pathway, thereby promoting angiogenesis and neurological recovery following SCI (Peng et al., 2021).

ROS and reactive nitrogen species (RNS), key factors in oxidative stress, mostly exist in the form of free radicals and are associated with the pathogenesis of common neurodegenerative diseases including Alzheimer’s disease, Huntington’s disease and Parkinson’s disease (Lin and Beal, 2006; Maiuri et al., 2019). The excess ROS induced by SCI produces peroxynitrite, which destroys mitochondria *via* carbonylation peroxidation, ultimately leading to the loss of neuronal function (Visavadiya et al., 2016). Crucial transcription factors including NF-ĸB, P53, AP-1 and Nrf2 are under regulation of ROS (Ohl et al., 2016). Oxidative stress-mediated inflammation is regarded to be a significant pathogenesis of SCI. Activation of NADPH oxidase produces excessive ROS, which induces phosphorylation of p38MAPK and JNK. Subsequently, NF-κB p65 is phosphorylated to elicit inflammatory responses. A novel marker of oxidative stress named advanced oxidative protein products (AOPPs) induces microglia-mediated neuroinflammation post-SCI through up-regulation of NADPH oxidase-dependent ROS and activation of MAPK/NF-κB signaling pathway (Liu et al., 2020a; Liu et al., 2021). Furthermore, BV2 cells with AOPPs can also result in pyroptosis through stimulation of NLRP3 inflammasome and induction of gasdermin-d cleavage (Liu et al., 2020a; Liu et al., 2021). Specific herbal molecules such as kaempferol are implicated as therapeutic agents to treat SCI, targeting oxidative stress and inflammation caused by the ROS-dependent MAPK/NF-κB and pyroptosis signaling pathway. Kaempferol, mainly derived from the rhizome of Kaempferol Galanga L, can inhibit the production of ROS through suppression of NADPH oxidase 4, thereby inhibiting MAPK/NF-κB pathway and pyroptosis (Liu et al., 2021). TLR4/NF-κB signaling pathway exerts crucial effects on multiple antioxidant and anti-inflammatory functions of certain herbal molecules. For example, catalpol may regulate HMGB1/TLR4/NF-κB signaling pathway by up-regulation of miR-142 to exert anti-inflammatory, anti-apoptotic and anti-oxidation effects after SCI in mice, thus promoting neuroprotection and functional recovery of spinal cord (Xia et al., 2020). Likewise, emodin is also one of the main bioactive components of several Chinese herbal medicines with anti-inflammatory effects (Alisi et al., 2012). Zeng et al. demonstrated that emodin inhibited oxidative stress and inflammation after acute SCI by activating Nrf2-ARE pathway, which down-regulates NF-κB to suppress the expression of IL-1β and IL-6 (Zeng et al., 2018). Apocynin, an antioxidant natural organic compound, inhibits gestational diabetes mellitus-induced inflammatory responses and oxidative stress in mice *via* suppressing TLR4/NF-κB signaling pathway (Liu et al., 2020b).

Taking all these studies into consideration, TLR4-NF-κB signaling pathway has a crucial impact on a variety of pathophysiological mechanisms of SCI, such as inflammation, autophagy, apoptosis, ferroptosis and anti-oxidative effects. Certain herbal drugs with their biomaterial carriers, which target TLR4-NF-κB signaling pathway, are potential therapeutic agents for SCI. Revealing diverse mechanisms of action and multifunction of these biomaterials and drugs can develop a more profound understanding of the pathophysiology of SCI and provide new ideas for more practical treatment approaches.

## 3 Empirical SCI Treatment Involving Biomaterial-Based TLR4-NF-κB Signaling Pathway

It is evident that regeneration of CNS will not be addressed through treatments based on a single surgery or drug, but through combinatorial therapies that simultaneously address the various injuries associated with SCI. Thus, engineered biomaterials are expected to exert crucial effects on treating secondary injuries to spinal cord by virtue of their ability to integrate multiple functionalities. In addition, increasing experimental models *in vitro* and *in vivo* have implicated that the TLR4-NF-κB signaling pathway is integral to many targeted injection strategies based on biomaterials. Here, we have classified these strategies according to different types of biomaterial and discuss the internal mechanisms of SCI treatment, including the modulation of TLR4-NF-κB signaling pathway, as shown in Table 1.

**TABLE 1:** Biomaterial strategies for SCI involving TLR4-NF-κB signaling pathway.

Type	Material	Therapeutic delivered	Functions	References
Hydrogel	Photo-crosslinked hydrogel	CSF1R inhibitor (PLX3397)	Reducing CD68-positive microglia/macrophages and mRNA levels of pro-inflammatory factors	Ma et al. (2020)
		Doxorubicin and TLR4 antagonist resatorvid (TAK-242)	Down-regulating TLR4 levels in 4T1 and RAW264.7 cells, suppressing NF-κB activation	Wang et al. (2021c)
	Hepatic acellular matrix (HAM) hydrogel	—	Promoting differentiation of macrophage M2 (CD68/CD206) through the TLR4/NF-κB signaling pathway	Li et al. (2021a)
	Chitosan/alginate hydrogel	Curcumin	Targeting macrophage, mitigating inflammatory responses through the TLR4-MAPK/NF-κB signaling pathway	Xu et al. (2022)
	Self-assembled supramolecular hydrogel	Rhein	Effective binding to TLR4, dephosphorylation IκBα and Inhibition of nuclear translocation of p65	Zheng et al. (2019)
		Curcumin and NO	Suppressing ROS-related p38MAPK/NF-κB signaling pathway	Deng et al. (2018)
	Peptide modified adhesive hydrogel	MSCs-derived exosomes	Providing damaged nerve tissue with an extracellular matrix, reducing inflammation and oxidation, alleviating microenvironment post-SCI	Li et al. (2020a)
	IKVAV Peptide amphiphile (PA) hydrogel	Brain-derived neurotrophic factor	Effectively inhibiting inflammation and protecting axons	Hassannejad et al. (2019)
	Fmoc-grafted chitosan (FC)/Fmoc peptide (FI) hydrogel	Curcumin, endogenous SCs	Modulating the phenotype of inflammatory cells and promoting myelin regeneration	Luo et al. (2021a)
	Silk fibroin hydrogel	Curcumin	Enhancing sustained release and skin permeability, decreasing NF-κB, TNF-α and IL-6 expression	Mao et al. (2017)
	Triglycerol monostearate (TM) and propylene sulfide 120 (PPS120) hydrogel	Curcumin	Down-regulating TNF-α, IL-1β and IL-6, reducing ROS levels, promoting nerve regeneration	Qian et al. (2021)
	High molecular weight hyaluronic acid (HMW-HA)	—	Binding to CD receptors, suppressing LPS-mediated microglial activation through modulation of TLR4 activation	Chistyakov et al. (2019)
Natural biomaterial	Bioactive hyaluronic acid (B-HA)	—	Ameliorating LPS-induced inflammation in macrophages via inhibition of TLR4 signaling	You et al. (2021)
	Thioketal-embedded chitosan/hyaluronic acid nanocarrier	—	Responding to oxidative stress and PH	Sabourian et al. (2020)
	Thioketal-embedded chitosan/hyaluronic acid nanocarrier	Curcumin, quercetin	Killing glioblastomacells, alleviating inflammation and oxidative stress	Sabourian et al. (2020)
	Calcium pectin and hyaluronic acid modified lactoferrin nanoparticles	Nerve growth factor	Promoting peripheral nerve growth, retaining protein bioactivity in the transplanted mouse dorsal root ganglion	Luo et al. (2021b)
		Rhein	Relieving inflammation via the TLR4/MyD88/NF-κB signaling pathway	
	Collagen	Human umbilical cord-mesenchymal stem cells (hUCB-MSCs) or patients’ own bone marrow mononuclear cells	Mitigating glial scar formation, providing guidance and support for axonal regeneration along collagen fibers	Zhao et al. (2017)
				Deng et al. (2020)
	Biomimetic hierarchical intraflofibrillary mineralized collagen (HIMC)	—	Up-regulating IL-4, IL-10 and TNF-α, inducing polarization of CD68 + CD163 + M2 macrophages, recruiting host MSCs	Jin et al. (2019)
				Shi et al. (2018b)
	Silk fibroin	—	Phosphorylation of p65, IκBα and IKKα/β, activating NF-ĸB signaling pathway	Park et al. (2018)
	Silk fibroin-based nanofiber membrane	Curcumin and 5-fluorouracil	Inhibiting signal transducer and activator of transcription3 (Stat3) and NF-κB signaling pathways	Xie et al. (2018)
	Silk fibroin hydrolysates	—	Inhibiting mitogen-activated protein kinase (MAPK) and NF-κB signaling pathways	Chon et al. (2012)
	3D bio-printed collagen/silk fibroin scaffold (3D-C/SF)	NSCs	Intensifying axonal connectivity, facilitating an orderly connected neural network	Li et al. (2021c)
	Chondroitin sulfate	—	Mitigating inflammatory responses in chondrocytes, macrophages and synovial cells via inhibiting NF-κB	Stabler et al. (2017)
				Korotkyi et al. (2020)
	Fucosylated chondroitin sulfate	—	Binding to RAW-264.7 macrophages through TLR2/4 recognition and in turn activating NF-κB	Jiang et al. (2021)
	Chondroitin sulfate methacrylate (CSMA) hydrogel	NSCs	Ameliorating forepaw hypersensitivity, inhibiting astrocyte differentiation and fibroglial cell formation	Liu et al. (2019)
	Alginate	—	Enhancing phagocytosis of macrophages by up-regulating TLR4 and activating AKTkt/NF-κB and p38MAPK signaling pathways	Bi et al. (2017b)
	Gururonate oligosaccharide derived from oxidative degradation of alginate	—	Ameliorating LPS-induced inflammation in macrophages by inhibiting activation of NF-κB and MAPK signaling pathways	Zhou et al. (2015)
	Esterified alginate micelle	Curcumin	Down-regulating TLR4 and downstream pro-inflammatory factors, mitigating infiltration of macrophages	Wang et al. (2021d)
Nanomorphology	PLLA multichannel tube composed of nanofiber channel walls (NNCs)	—	Reducing infiltration of macrophage/microglia and scar formation	Sun et al. (2019)
	Carbon nanotubes (CNTs)	—	Releasing ROS, activating NF-κB, up-regulating TNF-α and IL-1β	Facciolà et al. (2019)
	PH-PPS-PEG self-assembled nanomicelles	Tilianin	Inhibiting TLR4-NF-κB signaling pathway, down-regulating IL-1 and TNF-α, reducing ROS	Wang et al. (2018)
	Nanostructured lipid carrier	Berberine	Decreasing TNF-α, cyclocythase-2 and iNOS expression via inhibiting HMGB1/TLR4/NF-κB signaling pathway, regulating autophagy and apoptosis	Gendy et al. (2021)
	Nanocomposite hydrogel	Alginate and eudradit nanoparticles	Maximizing ROS sequestration, suppressing inflammation by inhibiting the activation of TLR4 and NF-κB	Fan et al. (2019)
	Poly-lactide-co-glycolide (PLGA) nanoccarriers	Andrographolide	Attenuating inflammation by reducing TLR4 and NF-κB p50 expression	Kulsirirat et al. (2021)

### 3.1 Hydrogel

Hydrogel, a 3D polymer composite system consisting of water and cross-linked polymers, can imbibe large quantities of water or biological fluids (Li et al., 2020b; Jian et al., 2021). With its excellent stability, malleability, biocompatibility and sustained release, hydrogel is an ideal drug carrier for SCI treatment. Nowadays, various hydrogelscan be constructed by numerous natural hydrophilic polymers such as alginate, chitosan, hyaluronic acid (HA), gelatinand cellulose, as well as synthetic hydrophilic polymers including polyethylene glycol (PEG), poloxamer, polyvinyl alcohol (PVA), acrylic acid and its derivatives (polyacrylic acid, polyacrylamide, polymethacrylate, etc.) through a variety of physical or chemical cross-linking. The construction of hydrogel also presents a trend of further intelligence. Photo-crosslinking, dynamic chemical bonding, small molecule self-assembly, semi-interpenetrating networks, dual networks, and 3D printing all showcase their characteristic advantages (Liang et al., 2021). Meanwhile, the function of hydrogel has converted from a single function such as molecule encapsulation or drug delivering to a multi-function combination (Liang et al., 2021). Figure 3 shows the strategies of hydrogel for SCI repair.

#### 3.1.1 Regulation of Microglia/Macrophage Polarization

**FIGURE 2:**
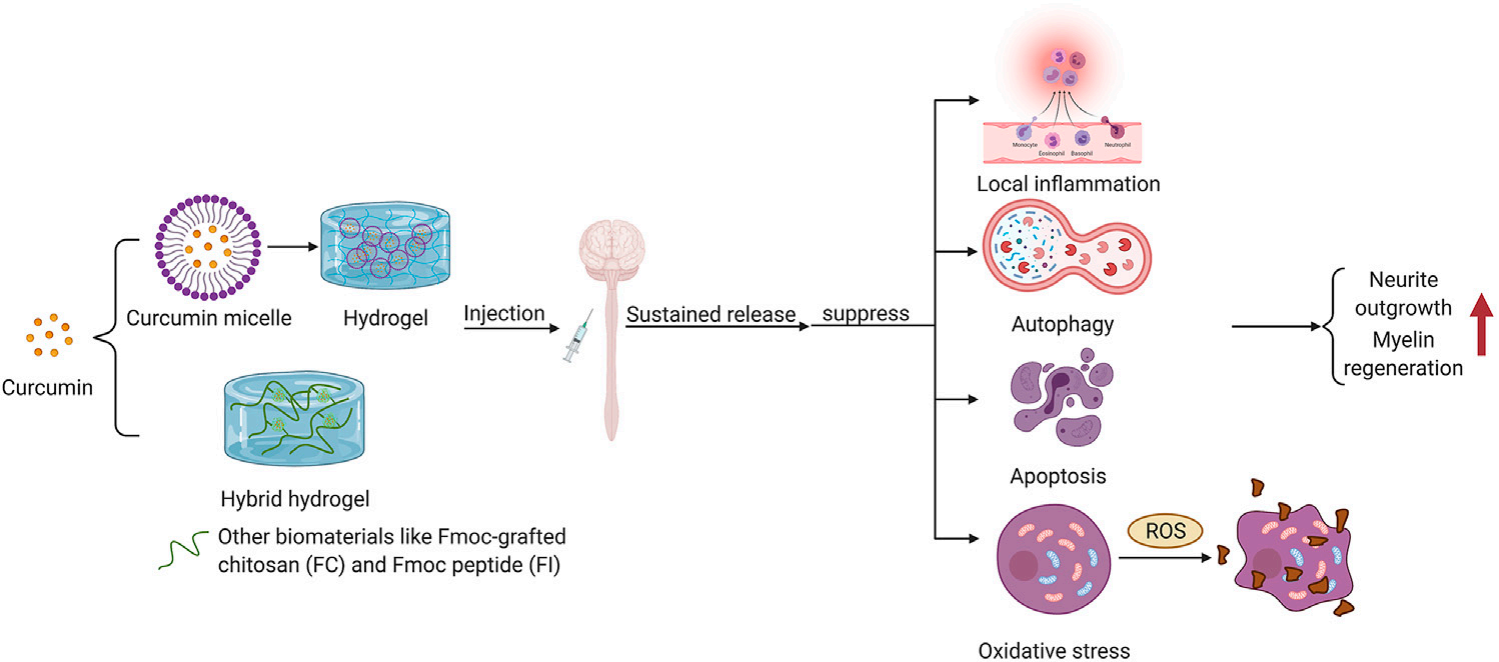
Hydrogel as a stent repair strategy for spinal cord injury.

Hydrogel-mediated tissue repair plays an important role in regulating microglia/macrophage polarization. A recent study evinced that photo-crosslinked hydrogel transplantation combined with colony-stimulating factor 1 receptor (CSF1R) inhibitor (PLX3397) can replace long-term activated microglia/macrophages through cell exhaustion and regeneration to reduce inflammatory responses mediated by microglia/macrophage post-SCI in rats (Ma et al., 2020). This novel combined therapy of hydrogel-based microglia/macrophage repopulation to repair complete transection SCI remarkably reduces CD68-positive microglia/macrophages and inflammation level, and also promotes the production of TUJ1-positive neurons in the lesion site (Ma et al., 2020). Furthermore, tissue-specific hydrogel can alleviate hepatic ischemia/reperfusion injury (IRI) in mice by modulating TLR4/NF-κB mediated polarization of macrophage. Injectable acellular matrix hydrogels have been shown to promote repairment in a variety of tissues. Lee et al. found that hepatic acellular matrix (HAM) hydrogels, which support the survival, repopulation and attachment of hepatocytes *in vitro*, promote the differentiation of macrophage M2 (CD68/CD206) through TLR4/NF-κB signaling pathway to ameliorate hepatic IRI (Li et al., 2021a) (Figure 2A). Anti-inflammatory herbal molecules-loaded hydrogel targeting macrophages also has a good therapeutic effect on macrophage-activated inflammation. Xu et al. fabricated curcumin-loaded chitosan/alginate hydrogel (CA-Gel) administration strategy with dual pH and ROS sensitivity. Chondroitin sulfate, which displayed good macrophage targeting, encapsulated curcumin and combined with CA-Gel to form nanoparticles (Xu et al., 2022). These oral nanoparticles released curcumin into macrophages and mitigated inflammatory responses through the TLR4-MAPK/NF-κB signaling pathway in mice with ulcerative colitis.

#### 3.1.2 Stem Cell Transplantation

Transplantation of SCs is a hot spot of potential SCI treatment. The axons of SCs can promote the healing of injured tissue by bridging the injured site. The ability to differentiate into neuron like cells enables SCs to possess regenerative ability (Assinck et al., 2017; Liu et al., 2018b). Proliferation and differentiation of endogenous SCs significantly promote nerve regeneration in rats. However, transplantation of SCs heavily depends on the microenvironment formed by injury site and delivery materials. Hydrogel is an ideal delivery material for stem cell transplantation due to its excellent biocompatibility and biodegradability (Figure 2B). It can reduce the adverse mechanical, biochemical and immune stress experienced by SCs after implantation and improves cell survival. For example, HA-hydrogels are functionalized with arginine-glycine-aspartic acid and heparin to encapsulate neural progenitors derived from human pluripotent stem cells (hPSC) to facilitate their survival after CNS implantation (Adil et al., 2017). More and more interest has also been attached to the application of multipotent mesenchymal stromal cells (MSCs) combined with extracellular matrix (ECM) based HA-hydrogels in tissue engineering. Monocyte-derived macrophages, which are grown on HA-hydrogel scaffold containing MSCs, show significant changes in immunophenotype, with higher expression of CD206 and lower expression of human leukocyte antigen (HLA)-DR and CD16, which indicates the least overall inflammatory profile (Hanson et al., 2011) (Figure 2B). Therefore, such cellular-hydrogel therapy may play a potentially significant role in regulating inflammation. Furthermore, in addition to these basic properties of hydrogel, its permeability is also of vital significance for stem cell transplantation. The high permeability of hydrogel is largely dependent on the low proportion of small mesh in hydrogel due to the more possible interaction between small mesh and molecules that diffuse through the network. Yuan et al. designed a highly permeable network of hydrogel molecules composed entirely of DNA double strands, avoiding the formation of small networks and successfully repaired 2 mm of spinal cord space in Sprague-Dawley rats. Therefore, implanted SCs proliferated and differentiated at the injured site, promoting the regeneration of neural network and restoring the basic hind limb function (Yuan et al., 2021). This hydrogel system shows great potential in tissue regeneration applications.

In addition to differentiation, the paracrine effect of SCs also promotes SCI repair. In recent years, MSCs-derived exosomes (MSC-EXOs) combined with miRNAs have shown great therapeutic potential in SCI recovery, and hypoxic preconditioning can optimize their therapeutic effects due to the hypoxia microenvironment *in vivo* (Liu et al., 2020c). Recent experiments have demonstrated that TLR4 is the target downstream gene of multiple miRNAs from MSC-EXOs (Liu et al., 2020c; Jiang and Zhang, 2021; Nie and Jiang, 2021). Hypoxic exosomal miR-216a-5p regulates microglia polarization through the TLR4/NF-κB/PI3K/AKT signaling pathway, thereby alleviating inflammation post-SCI (Liu et al., 2020c). MiR-145-5p and miR-23b ameliorate pathological damage and inflammatory responses following SCI by inhibiting the TLR4-NF-κB pathway, and there is a negative feedback regulation between miRNA-145-5p and TLR4 (Jiang and Zhang, 2021). Currently, although there are few studies on the assembly of hydrogel and MSC-EXOs that act on TLR4-NF-κB signaling pathway, hydrogel is widely used in MSC-EXOs load. Local injection of human urine stem cell-derived exosomes (USC-EXOs) embedded in hydrogel enables USC-EXOs to cross the spinal cord blood-brain barrier and transport the contained angiopoietin-like proteins 3 to the spinal cord injury area to promote angiogenesis (Cao et al., 2021). Due to the unclear retention and release in damaged spinal cord, Li et al. proposed an innovative implantation strategy for MSC-EXOs, in which they was immobilized in a peptide modified adhesive hydrogel (EXO-pGel). Different from systemic administration, locally transplanted EXO-pGel can be effectively retained and sustained-released in the injured site, providing the damaged nerve tissue with an extracellular matrix, reducing inflammation and oxidation, thus comprehensively alleviating microenvironment post-SCI (Li et al., 2020a).

#### 3.1.3 Direct Self-Assembly of Natural Small Molecules

A great deal of tissue engineering pays much attention to the implantation of scaffolds with bioactive cues to improve spinal cord regeneration. A number of supramolecular hydrogels containing drug compounds have been successfully developed in the laboratory for anti-inflammatory (Li et al., 2013; Pappas et al., 2016), bacterial resistance, wound healing (Brown and Anseth, 2017) and tumor suppression (Hu et al., 2018; Wang et al., 2017b). Among them, hydrogels containing specific drugs inhibit inflammation *via* the TLR4-NF-κB signaling pathway. Direct self-assembly of natural small molecules into hydrogels is a promising pharmacotherapy for the treatment of neuroinflammation (Figure 2C). Numerous studies have shown that natural molecules including camptothecin, rhein and paclitaxel can form self-assembled hydrogel system after structural modification (Zheng et al., 2019). Hydrogels can easily enter cells to bind to TLR4, thereby delivering the contained drugs such as rhein to suppress the TLR4-NF-κB signaling pathway, which can in turn ameliorate neuroinflammation. Compared with equivalent free drugs *in vitro*, the self-assembled rhein hydrogel has long-lasting efficacy, less cytotoxicity and better anti-neuroinflammatory effects with excellent sustained release, biostability and reversible stimulus response. Zheng et al. proved that the rhein supramolecular hydrogel enters the cell more easily than the free drug (Zheng et al., 2019). It increases the aggregation of TLR4 to bind closely to the active site of TLR4. Rhein hydrogel significantly dephosphorylates IκBα and inhibits the nuclear translocation of p65 in LPS-induced BV2 microglia, thus inhibiting the TLR4-NF-κB signaling pathway to achieve an optimal anti-inflammatory efficacy, which fundamentally modifies the inhibitory inflammatory microenvironment post-SCI to promote axon regeneration and reduce adverse reactions (Zheng et al., 2019). In addition to anti-inflammatory effects, self-assembled hydrogels targeting NF-κB are also known to inhibit autophagy and apoptosis. A novel self-assembled hydrogel loaded with NO and curcumin inhibits ROS-associated p38MAPK/NF-κB signaling pathway by lowering ROS levels, thereby alleviating overstimulated autophagy and apoptosis-induced myocardial ischemia/reperfusion injury (Deng et al., 2018). Currently, there are relatively few studies on natural small molecule self-assembled hydrogels acting on TLR4-NF-κB signaling pathway, which is a novel idea for drug delivery targeting TLR4 in the future.

#### 3.1.4 Combinatorial Implantation

However, the effect of single-cue hydrogel delivery was unsatisfactory, possibly because of the complexity of hostile niches in the injured area. Composite hydrogel with multi-mode signalinghas attracted much attention in application (Figure 2D). Man et al. designed a multifunctional nanofiber composite hydrogel AFG/fSAP composed of an aligned fibrin hydrogel (AFG) and a functional self-assembly peptide (fSAP) to promote spinal cord repair through a multi-mode signaling strategy mediated by heterogeneous cells (Man et al., 2021). AFG/fSAP, as a source of biochemical and biophysical signals, acts synergistically with multiple cues to promote spinal cord regeneration and functional recovery through acceleration of axonal regeneration and re-myelination, and promotion of tissue regeneration and angiogenesis. In addition, the combination of therapeutic molecules and growth factors with hydrogel scaffolds is a promising combinatorial approach to restore spinal cord function. And such combined implantation has a dual effect, not only promoting neuronal regeneration, but also effectively inhibiting inflammatory responses post-SCI. The photocrosslinked hydrogel loaded with CSF1R inhibitor (PLX3397) transplantation mentioned above can replace activated macrophage/microglia through cell regeneration, thereby inhibiting the expression of inflammatory factors (Ma et al., 2020). Wang et al. also fabricated a photocrosslinked hydrogel (D/T gel) combined with doxorubicin (DOX) and TLR4 antagonist resatorvid (TAK-242) simultaneously (Wang et al., 2021c). In a postoperative recurrence model of 4T1 murine mammary cancer, DOX and TAK-242 were slowly released by D/T gel at the tumor site. Both of them down-regulated TLR4 levels in 4T1 and RAW264.7 cells and suppressed NF-κB activation, thereby inhibiting the inflammatory microenvironment induced by the TLR4-NF-κB signaling pathway after surgical trauma and chemoimmunotherapy. In another study, the mixed implantation of nanofilm IKVAV biofunctionalized peptide amphiphilic hydrogel with BDNF protected axon growth and significantly inhibited inflammatory responses over a long sustained release time (Hassannejad et al., 2019). In terms of composite with other biomaterials, metal nanoparticles such as gold, silver, copper and zinc can also be used as biomaterials in combination with hydrogel to play antibacterial activities (Liang et al., 2021). For example, silver nanoparticles (AgNPs) hydrogel has excellent antibacterial activity to promote the wound healing of *staphylococcus aureus* infection (Haidari et al., 2021). Hydrogels can effectively control and optimize the delivery of silver to the wound, reducing the safety risk of AgNPs related to biotoxicity. In addition, AgNPs hydrogel also has important efficacy in the inhibition of inflammatory responses and apoptosis. In particular, silver can act synergistically with curcumin, which targets the TLR4-NF-κB signaling pathway (Varaprasad et al., 2011).

#### 3.1.5 Application of Curcumin-Hydrogel in TLR4 Signal

**FIGURE 3:**
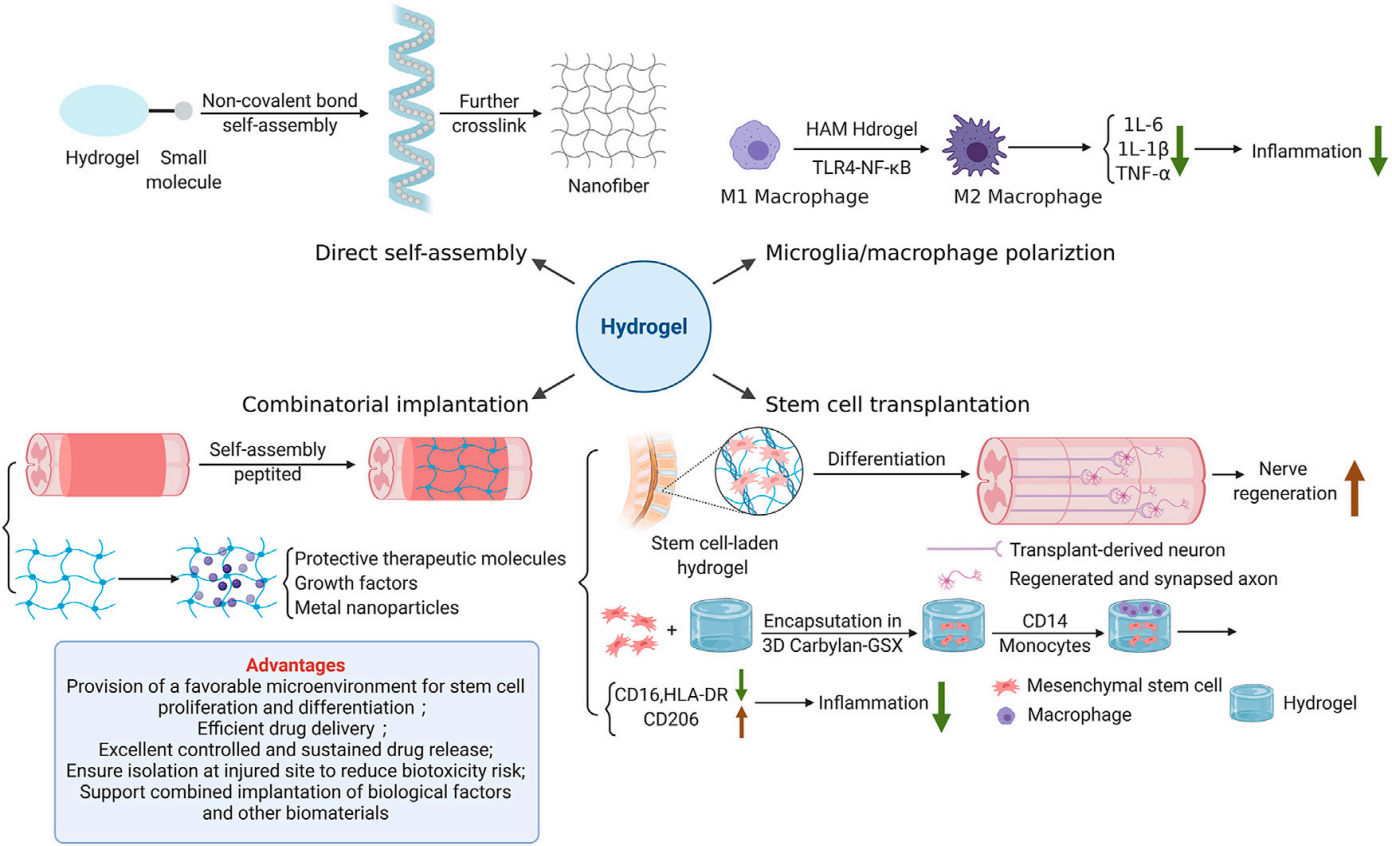
Schematic diagram of curcumin encapsulated in hydrogel for central nervous system injury.

Curcumin, a low molecular weight polyphenol compound that originates from rhizomes of Curcuma longa (Hasan et al., 2018), is a potential drug to promote wound healing with its excellent anti-inflammatory activity (Liang et al., 2021). Nevertheless, the therapeutic effects of curcumin are weakened by its poor biostability and water solubility. A number of experiments have shown that encapsulation of curcumin in composite hydrogel dressings can significantly improve its bioavailability. Wathoniet et al. proved that curcumin combined with 2-hydroxypropyl -γ -cyclodextrin hydrogel could improve the solubility and stability of curcumin while maintaining its antioxidant activity (Wathoni et al., 2017). In addition, the combination of curcumin and hydrogel is often accompanied by the application of nanotechnology. It has been revealed that the combination of the prepared curcumin micelles into hydrogels enhanced the encapsulation rate, drug loading and sustained antioxidant effect of curcumin (Liang et al., 2021). Li et al. found that loading the synthesized nano-curcumin to carboxymethyl chitosan/oxidized alginate hydrogel could significantly enhance the bioavailability of curcumin (Li et al., 2012). Applying Fmoc peptide (FI) and Fmoc-grafted chitosan (FC) as raw materials, Luo et al. developed a hybrid hydrogel named FC/FI-Cur hydrogel, which had persistent and slow release of curcumin and showed excellent injectability and self-healing properties due to the reversible π-π stacking of fluorenyl rings (Luo et al., 2021a). FC/FI-Cur hydrogel can accelerate neurite outgrowth post-SCI in rats and participate in the regulation of inflammation through modulating phenotype of infiltrated inflammatory cells, exerting outstanding effects in the remyelination process of regenerative nerves (Luo et al., 2021a). Figure 3 shows the multipathway therapeutic effects of curcumin encapsulated in hydrogel on central system injury.

Curcumin released by hydrogel exerts anti-inflammatory effects through the modulation of TLR4-NF-κB signaling pathway, and the suppression of IL-6, IL-8, IL-1β, TNF-α and cyclooxygenase-2 expression (Jurenka, 2009; Jin et al., 2014). Curcumin plays a neuroprotective role in ameliorating cerebral ischemia-reperfusion by regulating inflammatory and autophagy responses. Huang et al. revealed that curcumin potently enhanced neurological function in rats with middle cerebral artery occlusion by up-regulating phosphorylated AKT and mTOR and down-regulating TLR4, LC3-II/LC3-I, IL-1 and p38 (Huang et al., 2018). Meanwhile, it can also inhibit inflammation by modulating TLR4/p38/MAPK signaling pathway to exert neuroprotective effects (Huang et al., 2018). Mao et al. found that silk fibroin hydrogel permeated with curcumin embedded in polymer nanoparticles (CUR-NPs-gel) enhanced sustained release and skin permeability and significantly decreased NF-κB, TNF-α and IL-6 expression (Mao et al., 2017). Curcumin also inhibits pro-apoptotic inflammatory cytokines including ILs and TNF-α post-SCI in rats (Ruzicka et al., 2018). Yuan et al. revealed that curcumin effectively inhibited TGF-β, which acts as an upstream apoptosis receptor, and its inhibitory effects on apoptosis may be exerted after its regulation of TLR4-NF-κB (Ni et al., 2015), which is associated with inflammatory and apoptotic pathways (Yuan et al., 2019). A recent study manifested that curcumin ameliorates oxygen-glucose deprivation and reoxygenation-induced PC12 cell damage through suppressing C-C motif chemokine ligand 3 and inhibiting TLR4/MyD88/MAPK/NF-κB pathway to repress inflammation and apoptosis (Wang et al., 2020). Curcumin significantly suppressed the up-regulation of cytochromic C, caspase-3 and caspase-9, and decreased the number of apoptotic cells in a mice model of SCI (Feng et al., 2014). In addition, curcumin plays a significant role in regulation of oxidative stress. The study of neuroprotective effects of curcumin on white matter injury and its underlying mechanism, demonstrated that curcumin inhibited hypoxia-induced tissue damage and played a neuroprotective role through the crosstalk between Nrf2 and NF-κB signaling pathway (Daverey and Agrawal, 2020). It is noteworthy that curcumin has the most prominent inhibitory effect on ROS in the early stage of injury, and can significantly improve survival rate of astrocytes (Daverey and Agrawal, 2018). Nrf2 inhibits NF-κB activation by up-regulating HO-1 expression and preventing IκB degradation, thereby reducing ROS. Hydrogel-coated curcumin can be implanted *in situ* at the site of traumatic injury, effectively releasing curcumin, and significantly reducing ROS levels and inhibiting microglia activation. For example, curcumin-loaded hydrogel composed of triglycerol monostearate and propylene sulfide 120 has properties of post-traumatic microenvironmental responsiveness and ROS depletion (Qian et al., 2021). Deng et al. reported a novel self-assembled supramolecular hydrogel that could continuously release both curcumin and NO, down-regulating ROS levels and thereby suppressing ROS-related p38MAPK/NF-κB signaling pathway, effectively mitigating the ischemia/reperfusion damage (Deng et al., 2018).

### 3.2 Natural Strategies

Regulation of local cells and microenvironment exert critical effects on neuronal regeneration. Numerous studies have reported that biological scaffolds play an important role in fueling axonal regeneration post-SCI. Most of advances in biotechnology and bioengineering for SCI repair in recent decades are dedicated to the construction of biological support systems. The system is based on biomaterial application and is aimed at a favorable microenvironment for nerve cell repopulation and regulation of autophagy and apoptosis. Many natural biomaterials, including alginate, collagen and HA, which intensify not only cell attachment, growth, differentiation, but also interaction with peripheral nerve tissue. They are less likely to induce immune responses due to their excellent biocompatibility (Xie et al., 2018), thus can be utilized to alter the inhibitory microenvironment post-SCI. In addition, naturally derived materials are linked to the modulation of inflammation, autophagy and apoptosis after SCI *via* NF-κB signaling pathway.

#### 3.2.1 Hyaluronic Acid (HA)

Hyaluronic acid (HA), a major component of natural ECM of CNS tissues and NSCs, is a natural proteoglycan mostly found in eyes, skin and synovial fluid, and utilized in regenerative medicine and tissue engineering (Huerta-Ángeles et al., 2018; Garcia Garcia et al., 2020). HA plays a significant role in the transport of cells to CNS and is a good NSCs-carrier, enhancing the activity and differentiation ability of NSCs. Human embryonic stem cell derived neural stem cells (hESC-NS) encapsulated in HA hydrogel have been shown to promote differentiation into oligodendrocytes and improvement of motor function after SCI (Zarei-Kheirabadi et al., 2020). Luo et al. demonstrated that HA hydrogels provide a favorable microenvironment and structural support *in vitro* for bone marrow-derived mesenchymal stem cells (BMSCs) to grow, proliferate and eventually differentiate to neural cells (Luo et al., 2012). In terms of interaction with TLR4-NF-κB signaling pathway, bioactive hyaluronic acid (B-HA) fragments can ameliorate LPS-induced inflammation in macrophages through inhibiting TLR4 signaling. You et al. demonstrated that B-HA significantly raised IL-10 expression and inhibited the phosphorylation of p38, p65, IRF-3, JNK1/2, IκBα and IKKα/β (You et al., 2021). Low molecular weight hyaluronic acid (LMW-HA) and High molecular weight hyaluronic acid (HMW-HA) have distinct anti-inflammatory activities. The cellular signal transduction function of HA relies on the relative molecular weight. For a variety of myeloid cells including microglia, HMW-HA exhibits an anti-inflammatory efficacy and inhibits NF-κB activation through CD receptors such as CD44, while LMW-HA manifests pro-inflammatory effects. The effects of both are mainly reflected in TLR4 and TLR3 mediated reactions (Chistyakov et al., 2019; Bonet et al., 2020). However, the association between HA of different molecular weights and inflammation in these cells remains unclear (Chistyakov et al., 2019). HMW-HA is a polymer that can suppress LPS-mediated microglial activation through the modulation of TLR4 activation in microglia. Austin et al. showed that HMW-HA reduced the expression of LPS-mediated pro-inflammatory factors in microglia, including IL-6, IL-8, IL-1β and TNF-α, and decreased the expression of AKT and A20 proteins (Austin et al., 2012). In LPS-stimulated inflammation of mouse chondrocytes, LMW-HA induces a pro-inflammatory response *via* the up-regulation of TLR4, TRAF6, MyD88 and NF-κB expression in untreated chondrocytes, and enhancement of LPS effect (Campo et al., 2010). These findings implicate that the modulatory role of HA of any molecular weight on NF-κB activation may be determined by the crosstalk between HA and TLR4, and thus HA modulates inflammation through its different aggregation states (Campo et al., 2010). Chitosan/HA nanoparticles, which are stimuli-responsive and easy to assemble, are ideal for efficient delivery of small molecules to proteins under specific endogenous trigger conditions (Pornpitchanarong et al., 2020). Sabourian et al. reported a thioketal-embedded chitosan/HA nanocarrier with excellent encapsulation efficiency which could control the release of curcumin, quercetin and nerve growth factor (NGF) (Sabourian et al., 2020). Released curcumin and quercetin ameliorated TLR4-mediated inflammation and microglia-derived oxidative stress through the TLR4-NF-κB signaling pathway (Mohan et al., 2019; Le et al., 2020), and NGF-loaded nanoparticles promoted the growth of peripheral nerves and retained bioactivity of the protein in the transplanted mouse dorsal root ganglion (Sabourian et al., 2020). Calcium pectin (CP) and HA modified lactoferrin nanoparticles have been reported to encapsulate rhein (CP/HA/RH-NPs). CP layer enhances the stability of the complex and releases HA/RH-NPs to the lesion site, where HA/RH-NPs potently relieve inflammation *via* the TLR4/MyD88/NF-κB signaling pathway (Luo et al., 2021b).

#### 3.2.2 Collagen

Collagen, is a primary constituent of the natural extracellular matrix, is an insoluble fibrous protein (Khandaker et al., 2017; Benayahu et al., 2020). Collagen type I and II are widely applied to bone tissue engineering (Xing et al., 2021). Collagen type I has a repetitive structural motivation that is conducive to intermolecular or intramolecular assembly (Jang et al., 2021). When pH and temperature are within the physiological range, collagen molecules can self-assemble into a gelatinous state, creating a favorable microenvironment for cell growth (Jang et al., 2021). Scaffold-based strategies to construct regenerative microenvironment may represent a feasible therapeutic approach for patients with SCI (Zhao et al., 2017). Combined transplantation of NeuroRegen scaffolds and SCs has been proved to promote neurological regeneration in clinical studies of both acute and chronic complete SCI (Tang et al., 2021). Deng and Zhao et al. found that transplantation of human umbilical cord-mesenchymal stem cells (hUCB-MSCs) or patients’ own bone marrow mononuclear cells on collagen scaffolds can contribute to neurological recovery after acute or chronic SCI (Deng et al., 2020). When functionalized with various biomolecules, the collagen scaffold mitigates glial scar formation, provides guidance and support for axonal regeneration along collagen fibers and retains hUCB-MSCs at the lesion site to improve their effectiveness (Zhao et al., 2017; Deng et al., 2020). The modified collagen inhibits inflammation by regulating the polarization of macrophages. Jin et al. constructed biomimetic hierarchical intraflofibrillary mineralized collagen (HIMC) with nanointerface, which facilitated the osteogenic differentiation of MSC and attenuated inflammation by promoting the production of IL-4, IL-10 and TNF-α, and inducing the polarization of CD68 + CD163 + M2 macrophages (Shi et al., 2018b; Jin et al., 2019). Nevertheless, there are few applications of collagen in regulating TLR4-NF-κB signaling pathway in SCI repair, which is essential to understand the role of collagen in the treatment of SCI in bioengineering. Only one study suggested that jellyfish collagen activated both NF-κB and JNK through TLR4, thereby promoting macrophage production of TNF-α and IL-6 and participating in natural immune responses (Putra et al., 2014).

#### 3.2.3 Silk Fibroin (SF)

Silk fibroin (SF), a major component of silk, is a core silk protein with high biocompatibility and excellent mechanical and physiochemical properties (Rödel et al., 2018; Lv, 2020; Park et al., 2020). It is a promising natural material for biomaterial applications such as wound healing (Park et al., 2018). SF elicits activation of NF-ĸB through elevating IKKα, IKKβ and p65 levels and degrading IκBα, which in turn induces wound healing by regulating proteins including vascular endothelial growth factor, fibronectin, cyclin D1 and vimentin (Park et al., 2018). Therefore, NF-κB signaling is a promising therapeutic target for SF to be applicated in tissue engineering. Nevertheless, SF-related biomaterials is also likely to intensify inflammatory responses while applicated in the process of wound healing, thus it is potentially imperative to modify SF for the later amelioration of inflammation, which still remains to be solved (Xing et al., 2021). Xie et al. prepared a SF-based nanofiber membrane, which was loaded with therapeutic drugs (curcumin and 5-fluorouracil) and coated on polydioxane scaffolds for colorectal cancer treatment (Xie et al., 2018). Studies have confirmed that the system of combined-therapy drugs induces apoptosis of tumor cells through the inhibition of signal transducer and activator of transcription3 and NF-κB signaling pathways, having an obvious antitumor efficacy *in vitro* and vivo (Xie et al., 2018). Previous studies have demonstrated that SF indirectly suppressed osteoclast differentiation (Chon et al., 2012). Chon et al. found that SF hydrolysates inhibited MAPK and NF-κB, thereby inducing osteoclast apoptosis and having anti-osteoclastogenic effects (Chon et al., 2012). Notably, the combination of two or more natural biomaterials appears to produce synergistic effects on facilitating cell transplantation and nerve regeneration. For instance, Jiang et al. fabricated a 3D bio-printed collagen/SF scaffold (3D-C/SF) that mimics the normal anatomical structure of spinal cord. The implantation of 3D-C/SF combined with NSCs was confirmed to elicit a reduction in glial scar formation and acceleration of axonal regeneration (Jiang et al., 2020). Furthermore, Li et al. also manufactured a corticospinal tract structure of a 3D-C/SF stent implant, which significantly intensified axonal connectivity and facilitated an orderly connected neural network for the recovery of neurological function of transected SCI (Li et al., 2021c). Therefore, 3D-C/SF is a promising scaffold biomaterial for tissue repair after SCI.

#### 3.2.4 Chondroitin Sulfate

Chondroitin sulfate, similar to HA, is a polysaccharide widely distributed in ECM, synovial fluid and connective tissue, and usually attached to proteins as proteoglycans (Tobar-Grande et al., 2013; Vessella et al., 2021). As biomaterial of cartilage tissue engineering scaffold, chondroitin sulfate exerts important effects on homeostasis of articular joints (Tobar-Grande et al., 2013; Zimmermann et al., 2020). Under LPS stimulation, HA fragments significantly enhanceIL-1β expression, which can be suppressed by chondroitin sulfate (Stabler et al., 2017). The anti-inflammatory mechanism of chondroitin sulfate is that it acts upstream of the inflammasome to mitigate inflammatory responses in chondrocytes, macrophages, and synovial cells through inhibition of NF-κB activity, which is triggered by TLR4 or TLR2 agonists alone or combined with damage-related molecules (HA fragments) (Stabler et al., 2017; Korotkyi et al., 2020). Furthermore, modified chondroitin sulfate also regulates inflammation *via* TLR4-NF-κB signaling pathway. For example, fucosylated chondroitin sulfate, as a promising immunomodulator, can bind to RAW-264.7 macrophages through TLR4 and TLR2 recognition and in turn activate NF-κB, provoking immune function of macrophages to increase the expression of pro-inflammatory factors (Jiang et al., 2021). Dietary fucosylated chondroitin sulfate from Acaudina molpadioides (AM-CHS) inhibit both LPS production (*Escherichia coli*) and transcription of TLR4 and its downstream proteins, thereby mitigating chronic inflammation by decreasing the expression of pro-inflammatory cytokines and increasing IL-10 (Hu et al., 2019). In addition to inflammatory regulation, chondroitin sulfate is conducive to cell transplantation as a biocompatible biomaterial scaffold and the modification of chondroitin sulfate plays an important role. The combined implantation of chondroitin sulfate methacrylate (CSMA) hydrogel with NSCs can prevent or mitigate the adverse effects of NSCs transplantation for the treatment of SCI. Liu et al. found that transplantation of NSCs into 3D-CSMA hydrogel can ameliorate NSCs-induced forepaw hypersensitivity, suppress astrocyte differentiation and fibroglial cell formation, and promote nerve regeneration, thereby improving functional recovery after SCI (Liu et al., 2019).

Remarkably, it is a promising trend that composite hydrogel scaffolds composed of several natural materials are utilized in the application of biomaterials for drug delivery. Chitosan/alginate hydrogel loaded with curcumin is one such example, which alleviates inflammation by TLR4-MAPK/NF-κB signaling pathway (Xu et al., 2022). Furthermore, the micronano chitosan/chondroitin sulfate curcumin-loaded hydrogel formed by polyelectrolytic complexation is a novel kind of anti-tumor biomaterial. Polyelectrolytic complexation actively released chitosan, curcumin and chondroitin sulfate, and cytotoxicity studies manifested that polyelectrolytic complexation containing curcumin had an excellent anti-tumor efficacy, promoting apoptosis of HeLa cancer cells (Caldas et al., 2021). However, chitosan, HA, chondroitin sulfate and other natural polymers have poor mechanical properties, which affect the mechanical integrity of their composite scaffolds and impede their individual usage (Abbas et al., 2020). Therefore, some synthetic polymers with designed mechanical properties and controllable degradation rates, such as polyvinyl alcohol (PVA), polyglycolic acid (PGA), poly (lactic acid) (PLA) and poly (ε caprolactone) (PCL), are also widely used in drug deliver and neural tissue regeneration (Abbas et al., 2020).

#### 3.2.5 Alginate

Alginate is a natural hydrophilic polysaccharide derived from the cell wall matrix in marine brown algae (Inoue and Ojima, 2019; Manzari-Tavakoli et al., 2020). With favorable biodegradability and biocompatibility, alginate is an ideal cell encapsulation biomaterial for cell immunoisolation, usually utilized for scaffolds, cell encapsulation and therapeutic drug release in biological tissue engineering. Gelation is often involved in cell encapsulation. Lim et al. first attempted encapsulation of islet tissue within alginate to promote functional recovery. The gelation process enables alginate to forma hard, thick layer, which enhances the stability of the enveloped tissue or cell and protects them from immune responses (Jang et al., 2021). Alginate can encapsulate a variety of cell types, such as neuronal cells, islet beta cells, mesenchymal stem cells and endothelial cells, intensifying cell activity and metabolic capacity, and preventing their exposure for recognition by immune cells (Jang et al., 2021). As to sustained release, a nerve bridge based on SF/alginate composite biomaterials has been used as scaffold to load nerve growth factor (NGFs) in the repair of SCI (Jiao et al., 2017). Alginate microspheres released NGFs to the lesion site, effectively providing protection for spinal cord tissue and supporting neuronal survival. This combination of natural biomaterials, sustained release of microspheres and neurotrophic factors represents an attractive therapeutic approach for SCI.

Furthermore, alginate can intensify TLR4-NF-κB-mediated phagocytosis of macrophages. Bi et al. proposed that alginate enhanced the phagocytosis of murine RAW264.7 macrophages by increasing TLR4 expression and activating AKT/NF-κB and p38MAPK signaling pathways, inducing the activation of macrophages (Bi et al., 2017b). Despite these advantages of alginate as biomaterial, alginate has different pharmacological activities. Commercial crude alginate is likely to contain PAMPs that can be recognized by TLRs and elicit strong inflammation when implanted in rats (Paredes-Juarez et al., 2013). Nevertheless, alginate can be modified to exert anti-inflammatory effects. The esterified alginate-curcumin micelle, which significantly improved the hydrophilicity and bioavailability of curcumin, had obvious anti-inflammatory effect on RAW-264.7 cells (Wang et al., 2021d). Alginate-curcumin micelle enabled the commensal flora released by curcumin to effectively suppress TLR4 expression and downstream pro-inflammatory factors, and reduce the infiltration of macrophages, thus playing a targeted anti-inflammatory role in the treatment of ulcerative colitis. In addition, gururonate oligosaccharide derived from oxidative degradation of alginate potently ameliorates LPS-induced inflammation in RAW 264.7 cells through inhibiting NF-κB and MAP kinase signaling pathways (Zhou et al., 2015). These evidences implicate that modified alginate may be a promising anti-inflammatory agent for SCI treatment.

### 3.3 Nanomorphology

Nanoparticles can be processed into a variety of forms depending on its usage, such as liposomes, dendrimers, micelles and polymers (Junnuthula et al., 2021). Among them, the development and therapeutic application of nanopolymers (including nanoparticles, nanosilk, nanofibers and microbial nanobiopolymers) have been the focus of research in tissue engineering in recent years (Yang et al., 2019; Xing et al., 2021). Nanomolymers form polymer micelles during self-assembly. There are versatile options in shape, particle size, rigidity, charge, drug encapsulation and sustained release during assembly of nanomolymers (Junnuthula et al., 2021). Nanostructured conduits can be utilized in nerve regeneration, supporting scaffolds implanted at the site of injury and guiding axons from proximal to distal growth post-SCI. The micro-/nano-structure of implanted conduits can significantly suppress inflammation and scar formation at the lesion site. In a rat model of complete transected SCI, after a PLLA multichannel tube composed of nanofiber channel walls (NNCs) was transplanted, the infiltration of macrophage/microglia and scar formation were significantly reduced, and NSCs recruitment was accelerated (Sun et al., 2019). The ECM-mimicking nanostructures in NNCs facilitated the growth of nerve fibers in the channel, while the relatively dense nanostructures in the channel wall hindered the extension of nerve fibers (Sun et al., 2019). As conductive biomaterials for nerve repair, carbon nanotubes (CNTs) can be utilized to functionalize biomolecules and regulate cellular biological responses. As a kind of carbon-based material, CNTs have high electrical conductivity (105 S/cm) and large surface area, which can improve mechanical properties when combined with composites. However, in the CNS, CNTs may induce oxidative stress by releasing large amounts of ROS, and activating NF-κB, releasing TNF-α and IL-1β, and causing microglia-mediated inflammation (Facciolà et al., 2019).

Drug-loaded nanopolymers are widely used in regulating biological responses after SCI. The crosstalk between nanobiopolymers and macrophages can induce M1/M2 polarization of macrophages *via* TLR4-NF-κB signaling pathway to achieve pro-inflammatory or anti-inflammatory effects, thus regulating the microenvironment after SCI. Tilianin-loaded PH-PPS-PEG self-assembled nanomicelles (TLMS) inhibit the TLR4-NF-κB signaling pathway, and down-regulate IL-1 and TNF-α expression, thereby ameliorating macrophage/microglia-mediated inflammation and oxidative stress (Wang et al., 2018). Meanwhile, TLMS are highly efficient scavengers of ROS that inhibit the activation of caspase-3, thus preventing hypoxia/reoxygenation-induced damage to cells (Wang et al., 2018). Nano lipid carriers have also been verified as excellent carriers for drug-loading. Abdallah et al. developed a nanostructured lipid carrier of berberine (NLC BBR) that improved the bioavailability of berberine with anti-inflammatory properties. By inhibiting HMGB1/TLR4/NF-κB signaling pathway, NLC BBR decreased the expression of TNF-α, cyclocythase-2 and iNOS, and also participated in the regulation of autophagy and apoptosis, exerting protective effects on ischemia/reperfusion lesion (Gendy et al., 2021). In addition to the modulation of inflammation, the combined implantation of nanomaterials and hydrogel has significant effects on sustained drug release. Fan et al. fabricated a nanocomposite hydrogel loaded with alginate and eudradit nanoparticles, which improved stability and solubility of contained edaravone and maximized ROS sequestration, facilitating wound healing in diabetic mice (Fan et al., 2019). The alginate hydrogel promoted sustained release of edaravone, which could suppress the production of pro-inflammatory factors by inhibiting the activation of TLR4 and NF-κB (Fan et al., 2019; Wang and Lin, 2020). Poly-lactide-co-glycolide (PLGA) has been popularlyutilized as nanoccarriers owing to its favorable biocompatibility and biodegradability. Andrographolide (AG) is a renowned traditional medicinal plant that plays an anti-inflammatory role by inhibiting TLR4 and NF-κB p50 expression (Kim et al., 2018). When loaded by PLGA nanocarriers and subsepuently embedded in gelatine hydrogel, AG can have prolonged release and retention time in joints, preventing AG from being removed too quickly to achieve the desired therapeutic effect for osteoarthritis (Kulsirirat et al., 2021).

## 4 Conclusion and Prospects

The inhibitory inflammatory microenvironment, which results from a long-term complication of secondary injury after SCI, inhibits neuronal regeneration. TLR4-NF-κB is a classical implant-associated natural immunomodulatory signaling pathway. In this review, we reviewed the role of TLR4-NF-κB pathway in various pathological mechanisms of SCI and the functions of biomaterials in promoting spinal cord repair through NF-κB signaling. Activation of TLR4 and NF-κB up-regulates the expression of pro-inflammatory chemokines and cytokines, and participates in the regulation of autophagy, apoptosis, pyroptosis and ferroptpsis. Given that the activation mechanism of the TLR4-NF-κB pathway is well understood, it can be utilized to evaluate inflammatory responses post-SCI. Polarization of macrophages is highly regulated by various cytokines and bioactive substances induced by inflammatory responses after SCI, and its pro-inflammatory and anti-inflammatory balance (M1-M2 balance) can promote nerve repair, making it an ideal therapeutic target for treating inflammatory related secondary SCI. TLR4 induces activation of glial cells (astrocytes and microglia) in the spinal cord, leading to an increase in pro-inflammatory factors. Similarly, TLRs and NF-κB signaling also play important regulatory roles in autophagy/apoptosis—inflammatory pathways. Therefore, anti-inflammatory herbal medicine molecules (curcumin, etc.,) and natural biomaterials as drug carriers, both of which target TLR4/NF-κB signaling pathway, are potential therapeutic approaches to SCI. In addition, miRNA are also involved in the regulation of inflammation, oxidation and apoptosis *via* the TLR4-NF-κB pathway.

Due to the instability and insolubility of natural molecules, many novel drug-biomaterial complexes targeting TLR4 have been designed for cell encapsulation, delivery and sustained drug release. The synthesis of hydrogels, the application of natural biomaterials alone or in combination, and certain nano-physical surface modifications can potently promote the entrammel injection and drug release through TLR4 and NF-κB signaling, realizing more accurate and targeted release of bioactive molecules and regulation of inflammation. Meanwhile, hydrogels consisting of natural biomaterials facilitate the transplantation of NSCs, thereby promoting nerve regeneration. It is expected that future research will pay more attention to the development and application of novel composite biomaterials to facilitate the formation of an axon regeneration-favorable microenvironment at the injured site through known pathways. Among them, the TLR4-NF-κB signaling pathway ought to be one of the targets for SCI treatment in a controlled manner.
